# Surgical Treatment Strategies for Severe and Neglected Spinal Deformities in Children and Adolescents without the Use of Radical Three-Column Osteotomies

**DOI:** 10.3390/jcm13164824

**Published:** 2024-08-15

**Authors:** Pawel Grabala, Jerzy Gregorczyk, Negin Fani, Michael A. Galgano, Michał Grabala

**Affiliations:** 1 Department of Pediatric Orthopedic Surgery and Traumatology, Medical University of Bialystok Children’s Clinical Hospital, Waszyngtona 17, 15-274 Bialystok, Poland; 2 Paley European Institute, Al. Rzeczypospolitej 1, 02-972 Warsaw, Poland; 3 Medical Faculty, Medical University of Warsaw, 02-091 Warsaw, Poland; george.gregorczyk@gmail.com (J.G.); neginfani@gmail.com (N.F.); 4 Department of Neurosurgery, University of North Carolina, Chapel Hill, NC 27516, USA; mgalgano@email.unc.edu; 5 2nd Clinical Department of General and Gastroenterogical Surgery, Medical University of Bialystok Clinical Hospital, ul. M. Skłodowskiej-Curie 24a, 15-276 Bialystok, Poland; michal@grabala.pl

**Keywords:** severe scoliosis, neglected scoliosis, rigid scoliosis, adolescent idiopathic scoliosis (AIS), idiopathic scoliosis, scoliosis, spinal deformity, posterior spinal fusion (PSF), halo-gravity traction (HGT), temporary internal distraction rods

## Abstract

**Background**: Severe spinal deformity manifests as a pronounced deviation from the normal curvature of the spine in the frontal, sagittal, and horizontal planes, where the coronal plane curvature exceeds 90 degrees and may coincide with hyperkyphotic deformity. The most severe deformities exhibit rigidity, with flexibility below 30%. If left untreated or improperly treated, these deformities can result in serious complications associated with progression of the curvature. A combination of surgical techniques is frequently employed to attain optimal outcomes and minimize the risk of complications. The overall medical condition of the patient, their capacity to endure extensive procedures, the expertise of the surgeon, and the resources available all play significant roles in determining the course of management. A systematic and thorough review of the relevant literature was conducted utilizing a variety of electronic databases. The primary objective of this study was to scrutinize the surgical techniques commonly employed in complex spine surgeries for the management of severe scoliosis without resection vertebral body techniques, with higher potential risk of major complications, including permanent neurological deficit. **Conclusions:** Halo-gravity traction, halo femoral traction, and all techniques for releases of the spine (anterior, posterior, or combine), as well as thoracoplasty, have demonstrated significant effectiveness in managing severe and rigid idiopathic scoliosis. The combination of several of these methods can lead to optimal alignment correction without the need to perform high-risk techniques involving three-column osteotomies. Surgeons must customize the indications based on factors such as available resources, characteristics of the deformity, and the patient’s individual profile. Surgical correction of severe scoliosis without vertebral body resection surgeries decreases the potential risks related to neurological and pulmonary complications while providing significant clinical improvement outcomes. The powerful Ponte osteotomy is indicated for severe scoliosis, curves with poor flexibility, for better restoration of hypokyphosis, and decrease of hyperkyphosis. These corrective techniques combined with HGT or temporary internal distraction rods are recommended as viable options for managing individuals with severe rigid spine deformity characteristics. Therefore, they also should be considered and performed by a proficient surgical team. The presence of neuromonitoring is crucial throughout these procedures.

## 1. Introduction

Severe spinal deformity manifests as a pronounced deviation from the normal curvature of the spine in the frontal, sagittal, and horizontal planes, where the coronal plane curvature exceeds 90 degrees and may coincide with hyperkyphotic deformity [1,2,3]. The most severe deformities exhibit rigidity, with flexibility below 30%. Some researchers posit that rigid curvatures exceeding 80 degrees in the frontal plane and flexibility below 25% are important factors to consider. Nonetheless, it is crucial to acknowledge that these values serve as relative thresholds rather than definitive markers. It is worth noting that curvatures surpassing 70 degrees could already be deemed severe, rendering them challenging to address surgically due to the heightened likelihood of encountering potential complications [4,5,6,7,8,9,10,11]. Therefore, the correction of such curvature becomes significantly paramount in the overall management of the condition. It is imperative for healthcare providers to carefully evaluate the degree of curvature in order to determine the most appropriate course of action for optimal patient outcomes. In cases where the curvature exceeds certain critical thresholds, the decision to intervene surgically should be made judiciously, taking into account the risks and benefits associated with such procedures.

This deviation may manifest as functional abnormalities of the vertebrae, spinal cord stenosis, and back pain [2,3]. If left untreated or improperly treated, these deformities can result in serious complications associated with progression of the curvature [12,13,14]. In order to achieve the best possible correction, it is imperative to adequately mobilize this particular type of deformity—a process that often necessitates a more comprehensive surgical approach [14,15,16,17,18,19,20,21]. Special attention must be paid to preventing any clinical or neurological complications that may arise during the procedure. A combination of many surgical techniques is frequently employed to attain optimal outcomes and minimalize the rate of complications. The overall medical condition of the patient, their capacity to endure extensive procedures, the expertise of the surgeon, and the resources available all play significant roles in determining the course of management [21,22,23,24,25,26,27,28].

## 2. Material and Methods

A systematic and thorough review of the relevant literature was conducted utilizing a variety of electronic databases, including but not limited to EMBASE, PubMed, ScienceDirect, Web of Science, The Cochrane Library, Google Scholar, and Ovid MEDLINE. The exploration involved the utilization of a search strategy encompassing diverse combinations of keywords such as “severe scoliosis”, “severe spinal deformity”, “neglected scoliosis”, and “neglected spinal deformity”, incorporating both Mesh and non-Mesh terms relating to surgical treatments and outcomes. The most recent search was carried out on 31 May 2024, with additional pertinent studies identified through a meticulous manual examination of the references within the acquired publications. The inclusion criteria encompassed a variety of sources including original research studies, case reports, systematic reviews, and meta-analyses which had been formally documented in the English language for their abstracts. To prevent redundancy, in instances where a particular study group appeared in multiple literature pieces, the most recent publication was chosen for incorporation into the examination. Exclusions were enforced on standalone abstracts, articles that contained duplicated data, and those lacking unique research findings. Additionally, publications failing to meet the designated research criteria or lacking clearly outlined and transparent methodologies were excluded from the analytical process. Such stringent criteria were put in place to ensure the quality and reliability of the data being considered for the review.

The primary objective of this study was to scrutinize the surgical techniques commonly employed in complex spine surgeries for management of severe scoliosis without resection vertebral body techniques, considered as having a higher potential risk of major complications such as permanent neurological deficit [19,29,30,31,32,33,34,35,36]. The process of data extraction was conducted by a pair of autonomous authors, who meticulously collected data on a wide range of factors such as the geographical source, principal authorship, publication year, demographic characteristics of the study subjects, specifics of the interventions, research methodology, and the length of the monitoring period. In instances where discrepancies emerged during the data extraction phase, these were effectively addressed through interactive deliberations with the objective of achieving a shared understanding among all the authors engaged in the process. The collaborative efforts undertaken by the authors aimed to ensure a harmonious consensus and agreement on the information gathered, enhancing the reliability and robustness of the data analysis and interpretation.

## 3. Halo-Gravity Traction

**Figure 1:**
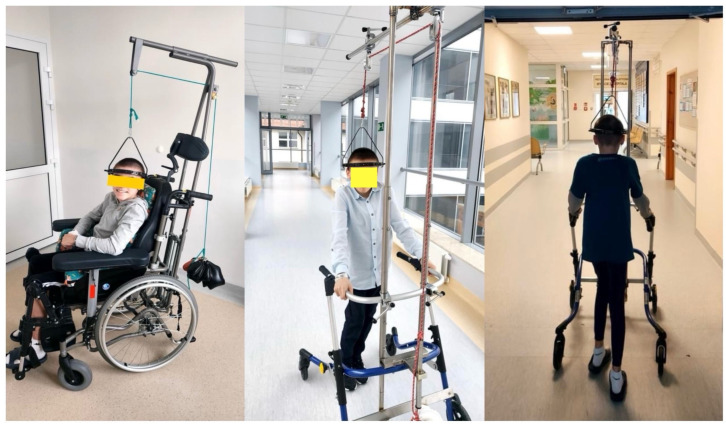
The clinical presentation of a 13-year-old individual suffering from severe kyphoscoliosis, who underwent pre-operative treatment with halo gravity traction lasting for a period of 3 months before proceeding with posterior spinal fusion.

Halo-gravity traction (HGT) is commonly utilized in clinical practice to mitigate the surgical risks in pediatric patients suffering from severe scoliosis prior to undergoing significant corrective procedures [37,38,39]. This method involves affixing a halo ring to the patient’s cranium in order to manage the advancement of the underlying spinal deformity [39,40]. The subsequent cephalalgia post-treatment is typically minor and endures for a period of 2–3 days. The procedure itself is generally painless for the pediatric patient, and research has indicated that children often experience a sensation of weightlessness during traction [41,42,43]. Application of the counterbalance is gradually increased until it reaches 50% of the total body weight. The duration of the traction regimen varies based on the extent of the deformity; nevertheless, the majority of patients undergo traction for a span of 3 weeks [34,37,41]. This method serves to elongate and align the spine, thereby enhancing its flexibility and lessening curvature prior to definitive surgery, consequently diminishing the surgical hazards and enhancing the overall outcomes. An example of a patient with severe kyphoscoliosis treated with pre-operative HGT is presented in Figure 1.

**Figure 2:**
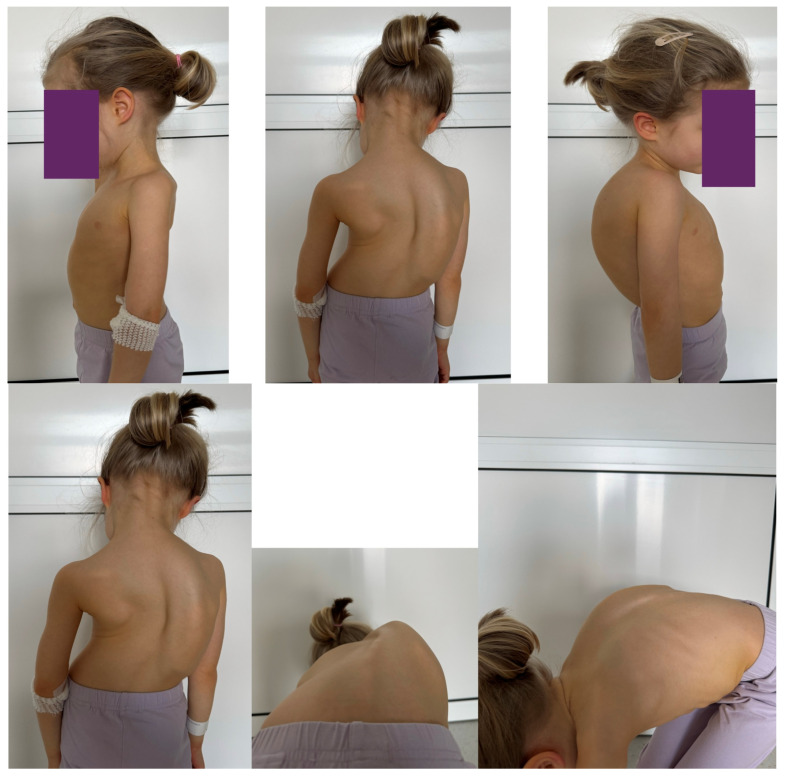

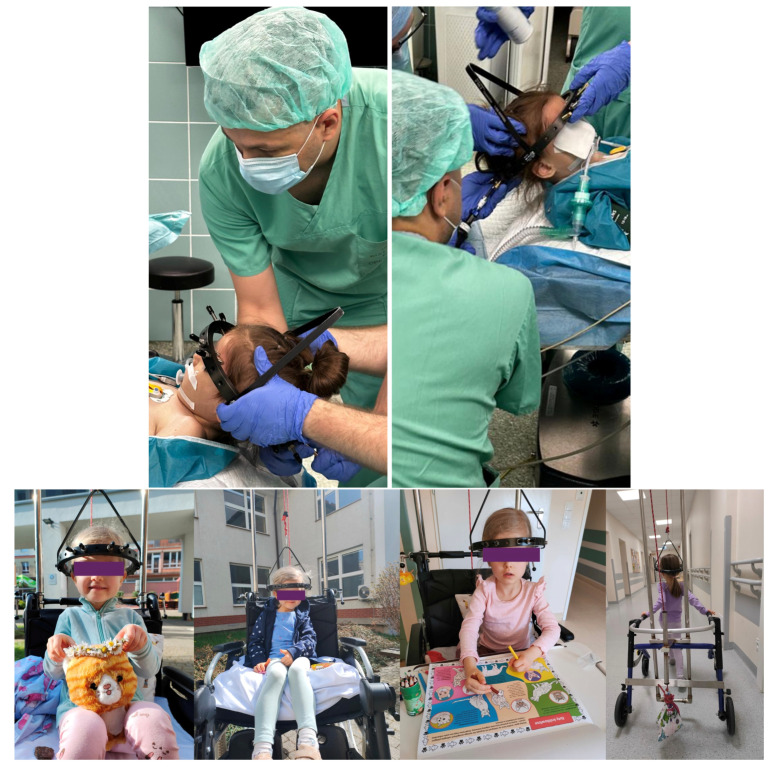
The clinical presentation of a 4-year-old girl with severe early onset idiopathic scoliosis, who underwent pre-operative treatment with halo gravity traction lasting for a period of 6 weeks before proceeding with Minimally invasive controlled growing rods placement.

Our approach to the treatment regimen of HGT entails the placement of the initial halo ring while the patient is under general anesthesia. The process of applying traction commences with a load of 2 kg and is incrementally augmented by 1 to 2 kg every 1 to 3 days, contingent upon the patient exhibiting adequate tolerance (with active traction maintained for at least 12 h per day). Additionally, 2–3 kg of weight is used at night-time as supporting traction, following guidelines from the literature [16,22,37,40,44]. There are many studies with different or similar protocols for the pre-operative treatment course [17,37,38,39,42]. The length of HGT therapy is established by considering the patient’s ability to adapt and tolerate the treatment, as well as potential pain control, whereby the traction force is gradually raised up to a maximum of 50% of the patient’s body weight [39,41,44,45]. According to the authors of this literature review, the eligibility criteria for initiating preoperative HGT involve the presence of curvatures exceeding 90 degrees in the coronal plane, alongside a flexibility measurement below 30% as assessed through bending films. Additionally, in situations where hyperkyphosis surpasses 90 degrees, it is recommended to utilize preoperative HGT. For cases of kyphoscoliosis with a flexibility level around 20%, the suggestion is to apply HGT, even when the primary coronal curvature ranges between 70 and 80 degrees. An essential consideration highlighted by the authors is the age of the patient, as younger individuals tend to exhibit better tolerance towards HGT, a factor of particular significance in early-onset scoliosis cases. The provided clinical images captures the procedure of applying the Halo ring under anesthesia in the surgical setting on a four-year-old girl with severe scoliosis of approximately 120 degrees as per the Cobb angle measurement, and treatment course (Figure 2).

**Figure 3:**
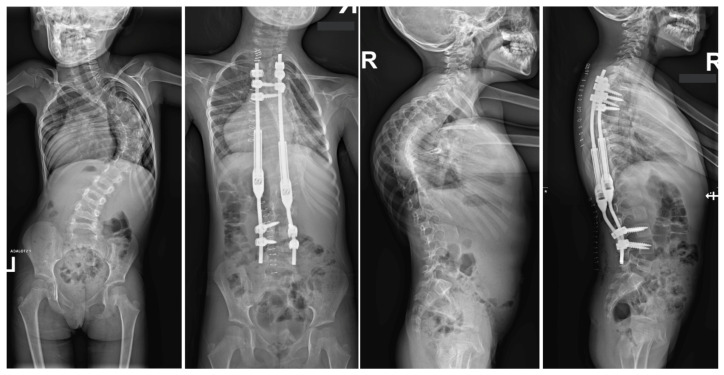

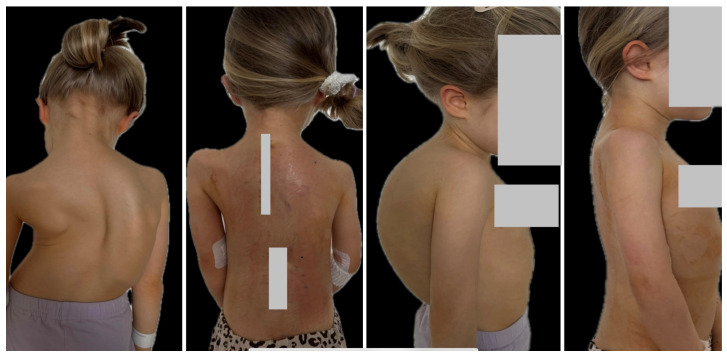
The X-rays and clinical images of a 4-year-old girl with severe early onset idiopathic scoliosis, who underwent pre-operative HGT and surgical proceeding with minimally invasive controlled growing rods placement [46].

Subsequently, the patient underwent a six-week preoperative HGT therapy following surgical treatment (Figure 3) and received minimally invasive controlled growing rods [46].

The authors suggest that the most favorable treatment duration with HGT is six weeks, although in instances where there is a noted enhancement in respiratory function and body mass, the traction therapy may be extended to three or even six months.

The intended outcome is the elongation and derotation of the spine [42]. A meta-analysis conducted by Sun et al. involving 210 patients revealed an average change in the coronal and sagittal Cobb angles of 63.36° and 49.66°, respectively, along with an average increase in height of 13.92 cm [47]. In another study, Garabekyan et al. [43] documented a 38% correction in the coronal Cobb angle and a 25% correction in the sagittal Cobb angle in their retrospective analysis. Additional notable benefits reported encompass favorable weight gain, enhanced pulmonary function, and respiratory capacity. Bogunovic et al. [39] established a positive association between halo traction therapy and improvements in pulmonary function test results. Similarly, Watanabe et al. [17] and Rocos et al. [38] demonstrated that halo traction yielded substantial enhancements in spinal deformities and pulmonary function, with only minor and treatable side effects. Nonetheless, some scholars have highlighted drawbacks such as challenges in daily activities, poor patient tolerance, the potential risk of cranial nerve impairments, cervical spondylosis, and paralysis [16,17,22,38,39,43,44,48]. Two studies noted occurrences of pin loosening, pin-related infections, and gastrointestinal discomfort [43,48]. Bogunovic et al. [39] reported a complication rate of 27%, which encompassed transient nystagmus, numbness in the upper extremities, pin site inflammation/infection, unilateral miotic pupil, and progression of myelopathy. In a separate study, Rocos et al. [38] identified that, of 24 patients, 1 exhibited early indications of cranial nerve dysfunction, 8 experienced pin loosening, and 1 displayed temporary urinary issues. Despite these challenges, halo gravity traction progressively facilitates spinal correction while diminishing neurological complications such as paralysis or nerve damage before and during the high-risk surgical procedure of corrective surgery with three-column osteotomies [20,47,49,50].

## 4. Halo-Femoral Traction

Halo-femoral traction (HFT) has been presented as an alternative for pre-operative traction, resembling the characteristics of HGT [51,52]. The utilization of HFT occurs prior to the definitive posterior instrumentation and fusion, with the possibility of a preceding anterior spinal release having taken place or not, prior to the application of traction [4,53,54,55]. In the available literature, an in-depth analysis of the HFT technique has been presented, incorporating an anterior spinal release into the procedure [47,48,51,52,56]. The initiation of HFT commenced two days subsequent to the anterior spinal release, with an initial weight of 1–2 kg, gradually escalating by 1 to 2 kg daily until reaching a percentage equivalent up to 50% of the patient’s body weight. The traction was maintained for a minimum of 12 h each day, being reduced by 50% during the patient’s sleep period. In cases of patients afflicted with idiopathic scoliosis, observations revealed that the major curve witnessed an average correction of 39% upon conclusion of the traction phase. This stands in stark contrast with the pre-traction side-bending radiographs, displaying an average correction rate of merely 24%. Similarly, patients with congenital scoliosis exhibited an average correction rate of 22% in pre-operative bending radiographs, which notably improved to 35% in post-HFT radiographs. It is worth noting that, unlike HGT, HFT is not transferable to devices such as wheelchairs or walkers, necessitating the patient to maintain continuous bed rest throughout the traction process [47,48,51,52,56]. The confinement to bed rest during traction poses a risk of heightened complications for the patient, such as pressure ulcers and pulmonary complications. This constraint often leads to more frequent removal of the traction device during the day, thereby limiting the overall duration of traction before proceeding to the definitive posterior fusion. Consequently, pre-operative HGT is generally preferred over HFT, considering these inherent challenges. The complications linked with HGT are similarly associated with HFT, with the added risk of brachial plexus palsy being linked to the latter [38,39,56]. Qiu et al. documented four cases where patients developed brachial plexus palsy during pre-operative HFT following anterior spinal release. Remarkably, all four patients experienced a complete recovery of function within a span of 2 months [57,58,59,60,61].

## 5. Intraoperative Halo Traction

**Figure 4:**
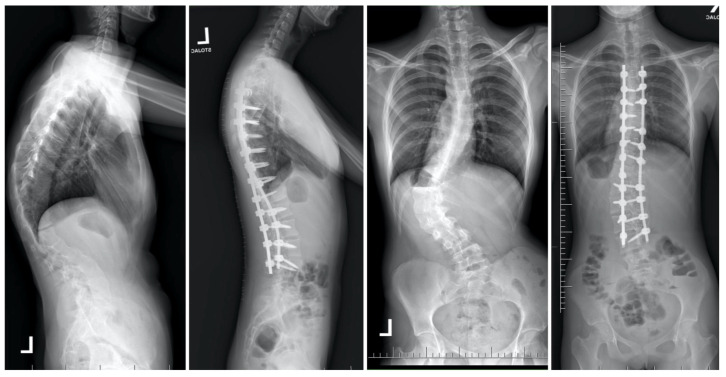
Radiographs taken before and after surgery at the last follow-up appointment depicting a 14-year-old female with severe adolescent idiopathic scoliosis who underwent intraoperative halo-femoral traction, followed by multi-level Ponte osteotomy and posterior spinal fusion in a single-stage surgical procedure.

Large spinal deformities, especially those originating from underlying neuro-muscular conditions or severe lumbar scoliosis, can pose a significant challenge when it comes to dealing with pelvic obliquity [62,63]. The presence of pelvic obliquity can lead to incorrect sitting posture and persistent pressure ulcers, making it a crucial issue to address for achieving a positive outcome; nevertheless, it is a task that is often intricate and multifaceted [63]. In the realm of addressing pelvic obliquity, intraoperative halo traction (HT) emerges as a valuable adjunct for improving the correction of this condition. A research study involving a cohort of 40 patients with neuromuscular scoliosis who underwent posterior spinal fusion with an extension of fixation to the pelvis highlighted that 20 of these patients were subjected to intraoperative HT [62]. The initiation of HT took place subsequent to the induction of anesthesia, with each halo being firmly secured by four pins. Following this, a sturdy Kirschner wire was carefully inserted through the distal femur on the side exhibiting the elevated hemipelvis. Upon assuming the prone position in the conventional manner, a traction of 6.8 kg was applied to the halo, which was then gradually escalated to an average of 11.3 kg until the pelvis achieved the desired alignment. The outcomes of this intervention indicated a notable 78% correction in pelvic obliquity within the HT group, in contrast to 52% in the control group [62]. Similarly, a detailed case report by Huang et al. illustrated the successful rectification of severe pelvic obliquity through the implementation of intraoperative HFT [63]. A separate study by Hamzaoglu et al. delved into the cases of 15 patients with thoracic scoliosis who underwent a treatment regimen involving intraoperative HFT in conjunction with posterior-only instrumentation [58]. Unlike prior analyses, HFT was not specifically employed for addressing pelvic obliquity in this scenario. The established protocol entailed obtaining a pre-operative traction radiograph while the patient was under anesthesia, resulting in an average enhancement of 51% in the major thoracic curve. In instances where the curve did not exhibit adequate correction, extensive facet resection and posterior release procedures were carried out. Noteworthy investigations focusing on intraoperative HFT have not documented any complications linked to traction [59]. Owing to the transient nature of the traction utilized, issues commonly associated with pre-operative traction, such as infections at the pin sites or loosening, are anticipated to be less prevalent in occurrence. Barsoum et al. chronicled a case involving an adult patient who was subjected to 2.3 kg of traction using Gardner-Wells tongs, and subsequently experienced a post-operative cranial nerve VI palsy [61]. However, reassuringly, this neurological impairment was completely resolved during the 6-month follow-up evaluation. Figure 4 shows X-rays of a 14-year-old girl treated with intraoperative halo-femoral traction.

## 6. Anterior Release

**Figure 5:**
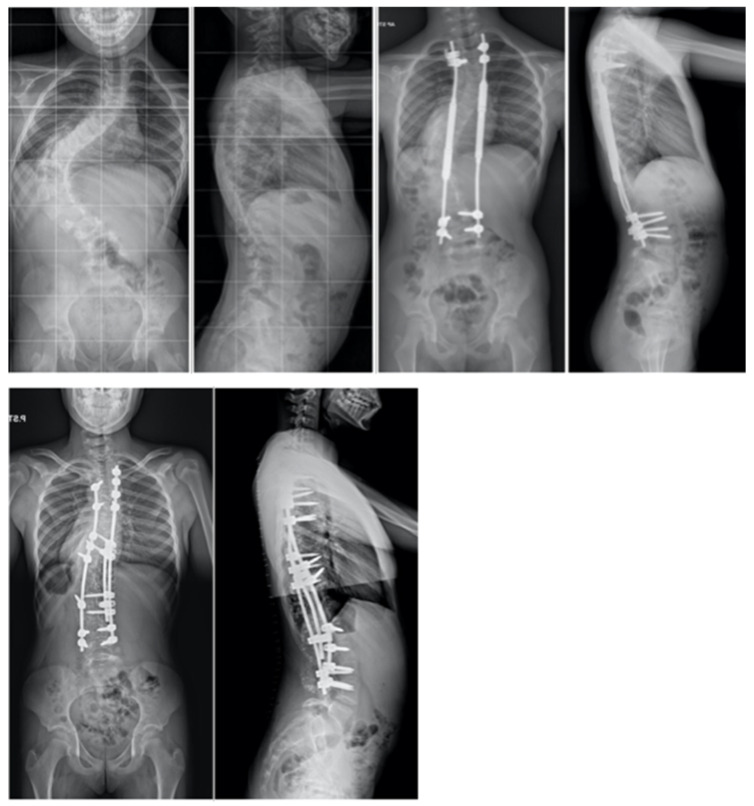
Radiographs revealed an 11-year-old female with severe early onset idiopathic scoliosis and asymptomatic spondylolisthesis. At the age of four, she underwent a four-level anterior release using a mini-open anterior approach, followed by the placement of magnetically controlled growing rods (MCGRs) through a less-invasive technique and subsequent periodic ambulatory lengthening. When she turned 11, she underwent a procedure involving conversion to posterior spinal fusion, replacement of MCGRs, and multi-level posterior release with Ponte osteotomy.

Anterior release procedures are often performed in the thoracic and lumbar spine to aid in increasing flexibility and correcting sagittal and coronal deformities, as mentioned in several studies [51,64]. These procedures, which involve anterior release and fusion, can be executed through either an endoscopic or open approach, with similar outcomes reported in the literature [51,64]. However, severe deformities present challenges due to anatomical changes in the chest wall and spine, making the endoscopic approach impractical in such cases. It is important to note that both the endoscopic and open methodologies have been associated with negative impacts on pulmonary function when compared to the posterior-only approach, a fact supported by various research studies [8,65,66,67]. In the thoracic spine, the surgical process typically includes excision of convex rib heads, removal of discs and posterior annulus, and the release of the posterior longitudinal ligament. Following this, the convex inferior endplate is often removed, sometimes along with the excision of the convex superior endplate, with or without the excision of the convex superior endplate, but the convex superior endplate is excised as well. Moreover, it is common practice to recommend anterior structural reinforcement in the lumbar spine and at the thoracolumbar junction to help prevent the development of kyphosis. After undergoing anterior release and fusion procedures, patients with severe and rigid curves may require instrumentation both anteriorly and posteriorly to enable a safe and effective three-dimensional correction of the spinal deformity [8,67]. The utilization of total pedicular constructs, with enhanced segmental fixation and improved capacity for tridimensional correction of AIS curves, has reduced the necessity for an anterior approach in specific curves. The anterior release technique encompasses the elimination of discs and rib heads near the apex of the curve, thereby enhancing spinal flexibility [8,66,67]. This procedure not only augments curve flexibility, but also ameliorates thoracic kyphosis during the subsequent posterior fusion. These adjustments are imperative for achieving appropriate post-operative spinal alignment in both sagittal and coronal planes. Following anterior release, patients typically undergo a secondary posterior spinal fusion to rectify scoliosis, which can be performed either in a single stage or in a staged manner [55,68]. Studies have reported that this combined approach achieves a correction rate of 40–50% in individuals with severe scoliosis [4,50,68,69,70]. Helenius et al. have asserted that at present, the primary indication for the anterior release technique is Lenke 5C thoracolumbar or lumbar adolescent idiopathic scoliosis curve, typically spanning levels T11 to L3 [71]. Controversy arises when comparing anterior–posterior release with other surgical methods, such as posterior-only release and anterior–posterior vertebral column resection, for the management of severe adult scoliosis. The principal benefits of combined anterior–posterior release encompass three-dimensional curve correction in severe and rigid idiopathic cases, reduced neuromuscular complications, infections, and pseudarthrosis [4]. Ren et al. discovered that anterior–posterior release is more effective than anterior–posterior vertebral column resection [49]. Some of the complications linked with anterior release include pseudoarthrosis and pulmonary issues [71]. Figure 5 shows X-rays of a 11-year-old girl treated with an anterior release, followed by the placement of magnetically controlled growing, and when she turned 11, she underwent a procedure involving conversion to posterior spinal fusion, replacement of MCGRs, and multi-level posterior release with Ponte osteotomy.

## 7. Posterior Release

**Figure 6:**
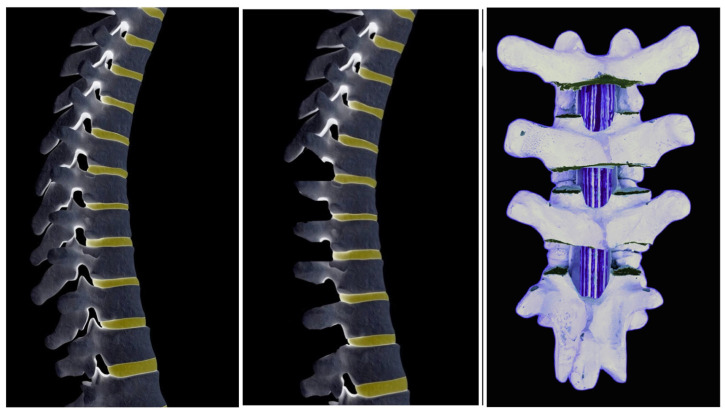
Ponte osteotomies involve posterior wide resection of superior and inferior facets, laminae, ligament flavum, and spinous processes.

**Figure 7:**
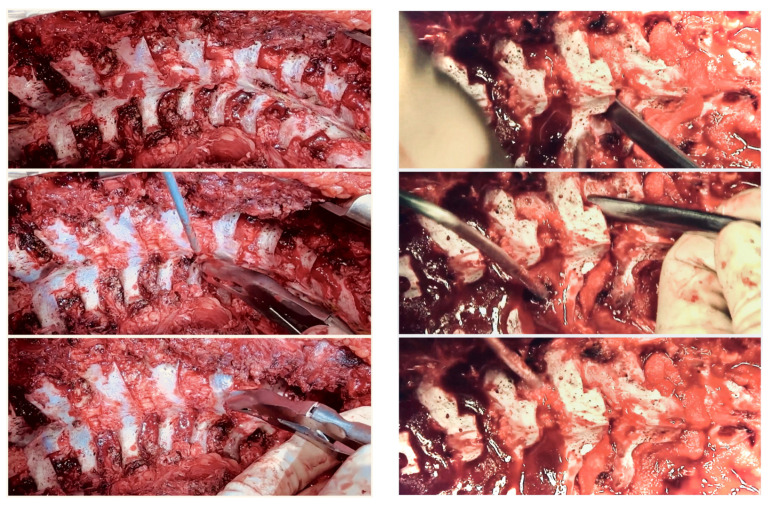
Intraoperative pictures of Ponte’s osteotomy technique: wide resection of superior and inferior facets, laminae, ligament flavum, and spinous processes.

**Figure 8:**
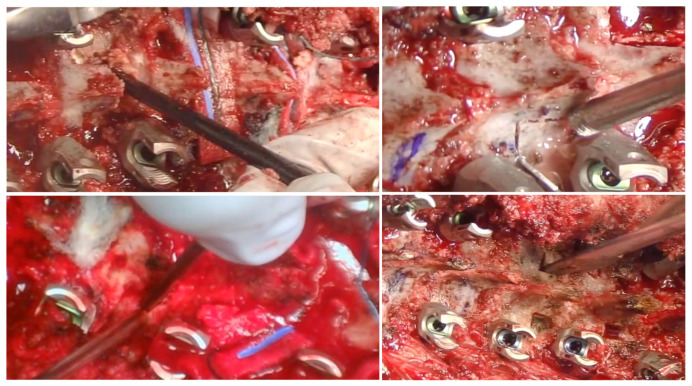
Intraoperative pictures of Ponte’s osteotomy technique when pedicle screws placed: wide resection of superior and inferior facets, laminae, ligament flavum, and spinous processes.

Severe scoliosis is characterized by bony changes that cannot be fully corrected through the release of soft tissues alone. When determining the appropriate osteotomy type to address this condition, several factors come into play. These factors include the extent of correction needed, the specific location of the deformity, the degree of imbalance in both the sagittal and coronal planes, the overall health status of the patient, and the expertise of the surgeon involved [19,26]. In numerous academic publications, the terms Ponte and Smith-Petersen osteotomy (SPO) are often used interchangeably, leading many surgeons to perceive them as identical surgical techniques. Despite this common misconception, it is essential to recognize that they are not entirely identical, and the resulting discrepancies need to be delineated and understood comprehensively. The discernible disparities between the two osteotomies manifest in various aspects, such as the type and extent of resections performed, the distinct anatomical locations for which they were originally devised (lumbar versus thoracic), and the classification according to Schwab’s Osteotomy Classification, with Grade 1 assigned to Smith-Petersen osteotomy and Grade 2 designated for Ponte osteotomy. SPO involves a precise resection of lumbar facet joints and the detachment of the ligamentum flavum from the inferior edge of the lamina and inferior articular process, without any resection of the laminae. When utilized for enhancing flexibility in thoracic kyphosis, the correction is achieved through a broad opening of anterior disc spaces and elongation of the anterior column. On the other hand, the Ponte osteotomy entails a broad resection of thoracic facet joints, laminae, and complete removal of the ligamentum flavum. In cases of thoracic kyphosis, the original indication, correction is facilitated by a significant reduction in the length of the posterior column, accomplished by closing the osteotomy gaps through segmentally applied and apically directed compression forces. The absence of lengthening in the anterior column resulting from extensive openings of anterior disc spaces helps maintain the immediate and long-term load-sharing capacity and stability of the corrective procedure. It is generally recommended to avoid using open-wedge osteotomies in the thoracic spine to prevent elongation of the thecal sac and the associated neurological risks. Ponte osteotomies, on the other hand, involve the posterior removal of superior and inferior facets, laminae, ligament flavum, and spinous processes to address scoliosis [19,26,72,73,74,75]. Figure 6, Figure 7 and Figure 8 depict the Ponte osteotomy release method.

**Figure 9:**
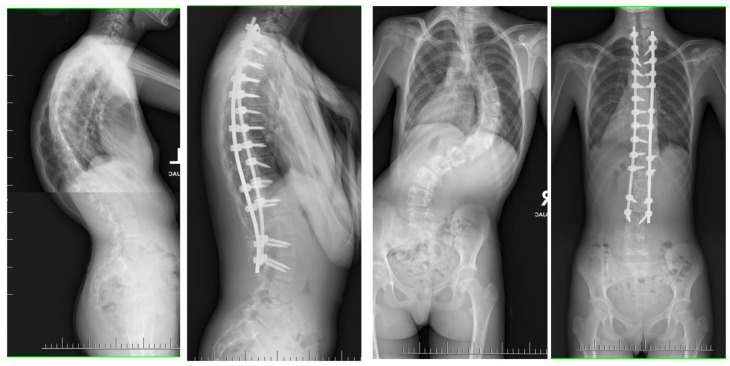
Radiographs obtained before and after the final follow-up examination revealed a 13-year-old female patient presenting with severe adolescent idiopathic scoliosis. The patient underwent treatment involving multi-level Ponte osteotomies followed by posterior spinal fusion in a single-stage surgical procedure.

Unlike SPO, Ponte osteotomy generally does not elongate or minimally elongates the anterior column of the spine, making it a safe option for thoracic spine procedures [19,26,72,73,74,75]. Thorough removal of laminae is imperative to mitigate the risk of spinal cord compression. Figure 9 shows a 13-year-old girl with severe scoliosis treated with multi-level Ponte osteotomies and following PSF.

Ponte osteotomies have been found to enhance sagittal kyphosis and enhance the three-dimensional derotation of the apex in individuals with AIS [74]. A study revealed that the incidence of IOM alerts was notably higher among patients who underwent Ponte osteotomies, compared to those who did not [75]. Despite these alterations, no patient experienced a heightened post-operative deficit. However, patients in the Ponte osteotomies group exhibited a higher rate of correction for coronal deformities and a greater kyphosis Cobb angle during post-operative monitoring [75]. Other research has indicated that both SPO-treated and Ponte osteotomy-treated patients had similar rates of surgical complications immediately after corrective surgery. Patients with severe rigid kyphoscoliosis and existing neurological deficits at baseline were more likely to experience neurological complications following corrective surgery [26,76,77,78].

Posterior column osteotomy (PCO) corrective techniques consist of SPO and Ponte osteotomy. They are recommended as viable options for managing individuals with severe rigid spine deformity characteristics [26]. While Ponte osteotomy enhanced the three-dimensional correction of AIS, it also led to an elevation in the incidence of IOM alerts in 12.5% of instances [26]. Ponte osteotomies are carried out in elongated non-angular hyperkyphotic thoracic deformities, regardless of whether they involve idiopathic scoliosis, rigid deformities, or proximal junctional kyphosis following instrumented fusions, as these conditions can benefit from the implementation of this technique, which necessitates a mobile anterior column to rectify the deformity. Ponte osteotomy, in conjunction with Smith-Petersen osteotomy, constitutes a posterior column osteotomy. The degree of correction may reach 10° per level if the intervertebral discs remain mobile [76]. Performed on multiple levels, it offers powerful opportunities to increase the mobility of the spine and thus influences the correction of the spine in three planes as a powerful surgical technique.

## 8. Temporary Internal Distraction Rods

**Figure 10:**
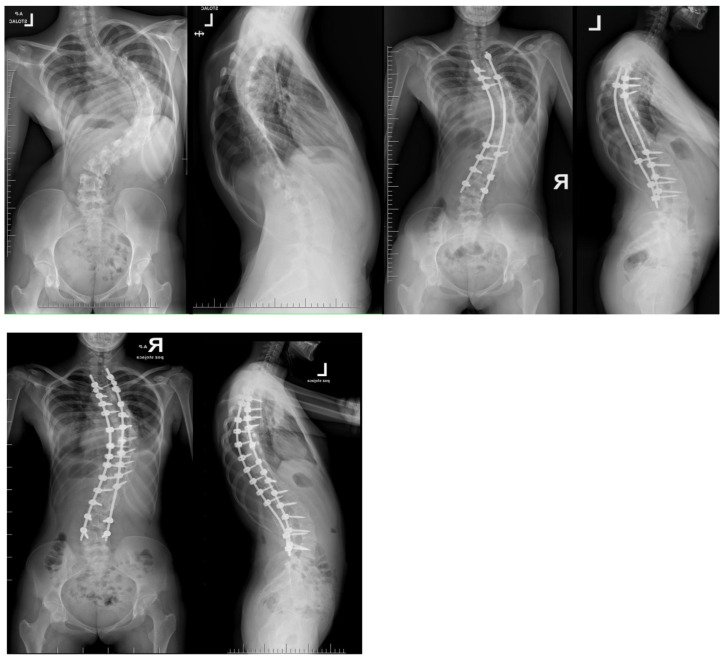
A 17-year-old female patient underwent a series of surgical procedures involving the less invasive temporary internal distraction technique followed by posterior spinal fusion. Radiographs were conducted before the initial stage, after the first stage, and post-operatively during the final follow-up.

Temporary internal distraction (TID) involves the placement of fixation points at the top and bottom of rigid spinal curves, utilizing spinal instrumentation to distract the spine akin to methods employed in growing rod constructs [18,22,23,33,69,79,80,81,82,83]. Due to prolonged hospitalization and potential complications linked to HGT and hybrid fixation techniques (HFT), these options may not be suitable for all patients [44,84]. TID could serve as a viable alternative in cases where external traction is not recommended and can also be integrated into a single-stage procedure to complement other corrective interventions [18,23,82]. A study involving 10 patients with significant, inflexible curves, for whom HGT was unsuitable, revealed that 6 patients underwent an initial anterior release, while 4 did not [18]. All patients received temporary posterior distraction instrumentation and subsequently underwent definitive fusion surgery approximately 2.4 weeks later. During treatment, six patients underwent multiple distraction procedures [18]. Buchowski et al. observed an average curve correction of 53% (ranging from 39% to 79%), exceeding the pre-traction bending radiograph correction and yielding comparable results to HGT and HFT [83,84,85,86]. The study reported no instances of neurologic or infectious complications [18]. In a separate investigation encompassing eleven patients afflicted with severe and inflexible scoliosis who underwent treatment with TID, Hu and colleagues documented a notable 53% amelioration in the major Cobb angle, concomitant with an enhancement in forced expiratory volume in 1 s from 61.4% to 71.3% [79]. Furthermore, the scholars observed the conspicuous absence of any neurologic or infectious complications arising from the procedure. The methodology employed for TID closely mirrors the technique delineated by Buchowski and colleagues [18,23], albeit with certain modifications, as elucidated in the existing literature [22,23,33,69,80,81,82,83]. The conventional prone positioning is utilized, consistent with the approach for any intervention involving the posterior aspect of the spine. The integration of neuromonitoring stands as a pivotal and indispensable phase, particularly throughout the distraction process. The initial stride entails effectuating a midline incision in the skin, succeeded by subperiosteal dissection to expose the requisite anchor points. For the cephalad fixation, either infralaminar or subpedicle hooks are situated. It is imperative to circumvent sitting these hooks at the designated levels for ultimate fusion to avert potential bone impairment. Additionally, cephalad anchor points might encompass the ribs. Typical caudal anchor points include down-going laminar hooks, lumbar pedicle screws at adjacent levels, or attachment to the pelvis. These anchor points frequently slacken during the distraction phase and should not function as ultimate anchor points for fusion. If iliac screws are employed, they should be positioned to accommodate the insertion of new screws just distal to them during the definitive procedure. Diverse rod constructs can be utilized, with the most elementary one comprising a rod for cephalad anchors and another for caudal anchors interconnected by a side-to-side connector. Subsequent to the placement of rods and the execution of distraction, extensive posterior releases are executed at each rigid deformity level. Gradual increments in distraction are subsequently applied to harness the viscoelastic characteristics of the spine and attain maximal distraction. The surgical incision is sutured according to the surgeon’s preference, and patients are mobilized postoperatively sans the necessity for bracing or casting [22,86]. Ordinarily, a minimum of one week of TID is permitted before definitive fusion is undertaken. Prolonging the distraction period beyond that is feasible but is improbable to yield enhanced correction. Throughout the definitive fusion, the provisional implant is extracted, and the ultimate instrumentation is introduced. TID can also be conducted in a single-stage modality. The distraction construct is positioned early in the procedure to instigate distraction, while other facets of the surgery are finalized. The construct can subsequently be gradually elongated until the final instrumentation is in situ. Subsequently, the TID construct is eliminated before closure, obviating the necessity for a subsequent procedure [22]. Hu et al. propounded an alternative strategy for treating thoracolumbar idiopathic scoliosis, entailing minimally invasive incisions specifically at the requisite anchoring levels [79]. Unlike other methods, the authors did not carry out sub-periosteal dissection. Their technique involved placing two pedicle screws at the top and bottom levels of the primary Cobb angle, connecting them with rods and a side-to-side crosslink. The authors advised post-operative orthosis use and allowed a distraction period of up to 15 weeks before proceeding with definitive fusion [22,79]. Figure 10 presents a 17-year-old female patient underwent a staged surgical treatment involving the less invasive temporary internal distraction technique followed by posterior spinal fusion.

## 9. MCGR as Temporary Internal Traction

The MCGR as a temporary internal traction method, as outlined by Di Silvestre et al. [83], involves a two-stage posterior correction process to effectively treat severe adolescent idiopathic scoliosis (AIS). Following the initial surgery, a gradual distraction using the MCGR resulted in an average lengthening of 2 cm over a period of 2 weeks without any complications. During the subsequent surgery, the MCGR was replaced with definitive rods for final fusion, with a mean pedicle screw density of 93.3%. The study authors concluded that the gradual traction method with MCGR for severe AIS is a safe approach for progressive curve correction prior to the final fusion, without the risk of neurological complications associated with more aggressive single-stage surgeries. In a staged manner, MCGR has been presented as a viable alternative to halo traction, effectively reducing the occurrence of traction-related complications [83].

**Figure 11:**
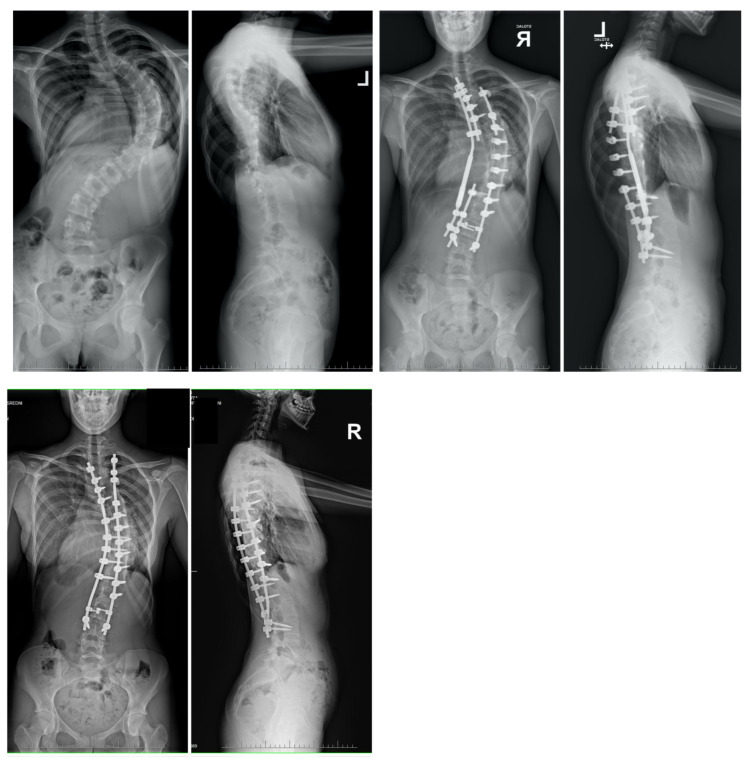
During the observation period, pre- and post-operative radiographs were utilized to monitor the treatment progress of a 16-year-old female diagnosed with severe adolescent idiopathic scoliosis. Staged surgery was conducted, involving a temporary internal distraction device with MCGR for initial intervention, followed by the implementation of double Co-chr 6.0 rods for final correction of the spinal curvature.

In the alternative approach delineated by Grabala et al. [44], patients were subjected to a two-phase surgical strategy. Initially, a comprehensive standard approach to the spine was carried out from the rear direction, enabling visualization of the spine at the specified stabilization levels. Facetectomy was performed at all spinal levels bilaterally, excluding the upper instrumented vertebrae (UIV). At all levels [73,74,75,76], a typical Ponte osteotomy was executed, with segmental screws being implanted at each level (at least on one side). A properly curved MCGR was positioned on the concave side of the curvature to achieve partial correction, while neuromonitoring guided the procedure. Pedicle screws on the convex side’s upper and lower levels were linked by a temporary short stabilizing rod, forming two firm blocks to strengthen the structure for future spinal distraction [44]. The patient was discharged after a five-day hospital stay, and weekly spinal distractions were performed over the next six weeks until reaching the maximum MCGR distraction point. Each distraction was carried out with maximum torque or until the observation of the “clunking phenomenon”. The forces applied during spinal distraction were of significant magnitude, and a single distraction control image was taken after six weeks of gradual lengthening of the magnetic rod. Subsequent to the completion of additional MCGR distractions, a final correction of the spinal deformity was conducted, involving the removal of the MCGR. The ultimate correction encompassed a blend of rod distraction/compression, apical translation, and segmental derotation. In the ensuing treatment phase, the resultant distraction length of the rod was measured using a ruler [44]. Figure 11 shows the treatment of a 16-year-old female diagnosed with severe adolescent idiopathic scoliosis with staged surgery, involving a temporary internal distraction device with MCGR for initial intervention, followed by PSF.

The study conducted by the authors of reference [44] concluded that the utilization of a magnetically controlled growing rod (MCGR) as a temporary internal distraction device for neglected scoliosis in adolescent patients does not present any significant advantages. However, it was found that surgery for severe scoliosis can be performed safely with lower risks of complications by implementing pre-operative HGT. An important intraoperative challenge associated with utilizing an MCGR as a temporary internal distraction device was the occurrence of a 50% probability of temporary neuromonitoring changes due to excessive force on the spine and substantial distraction. Nonetheless, both techniques yielded comparable clinical, radiographic, and pulmonary function outcomes. The use of HGT was shown to result in reduced blood loss and shorter anesthesia duration. The strategy of achieving partial correction was highlighted as crucial in preparing for subsequent interventions by gradually decreasing the curvature, thereby decreasing the surgical complexity and the likelihood of neurological complications as described in reference [34]. Another study by Koller et al. examined a similar method of temporary internal distraction utilizing MCGR for the management of severe scoliosis [81]. A group of seven patients with a primary curvature exceeding 100° underwent treatment involving temporary MCGR placement. The treatment protocol included posterior instrumentation, periapical release with advanced Ponte osteotomies, segmental pedicle screw insertion, and the use of a single MCGR. Subsequent to about 14 days, a second surgical procedure was carried out to remove the MCGR, achieve final correction, and perform fusion. Notably, none of the patients encountered major complications or neurological deficits. The staged surgical approach led to an average correction of the post-operative primary curve to 39° (67%). The ultimate conclusions of the study propose that this technique may have the ability to decrease the necessity for HGT and the high-risk 3 Column Osteotomies (3CO) in the correction of severe scoliosis as outlined in reference [81].

## 10. Rib Resection/Thoracoplasty

**Figure 12:**
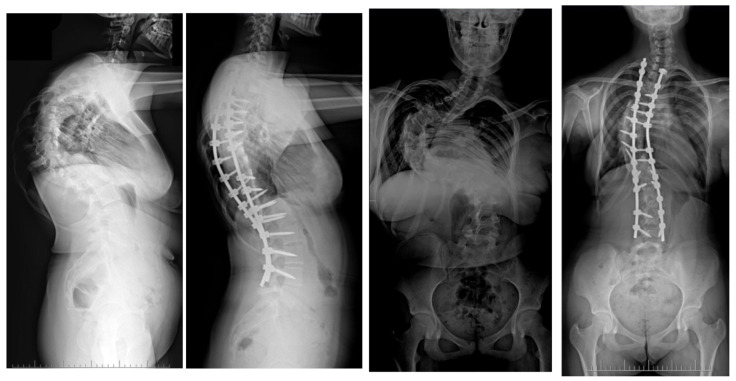
A 14-year-old female patient suffering from severe adolescent idiopathic scoliosis underwent treatment involving pre-operative halo gravity traction, followed by multi-level Ponte osteotomies, a 5-level rib resection with thoracoplasty procedure, and eventual correction using double Co-chr 6.0 rods. Radiological photographs were taken both before and after the surgical intervention, as part of the observation period.

An alternative surgical procedure has been developed to manage severe scoliosis characterized by noticeable shoulder and chest asymmetry [86,87,88,89,90]. A common accompanying feature is unilateral rib prominence, which has been connected to lower self-reported patient outcome scores and self-image scores in affected individuals [88,89,90]. Thoracoplasty (TP), initially designed for treating pulmonary tuberculosis, involves the removal of portions of the ribs to correct the deformed chest wall. This procedure typically includes excision of the affected ribs from the costovertebral junction to the posterior axillary line. Various adaptations of this technique exist, such as Schollner costoplasty, convex short-length rib resection, rib-end-to-transverse process thoracoplasty, and short apical rib resection [91,92,93,94]. The primary objective of TP in scoliosis patients is to address residual rib prominence, which can also decrease the rib hump height, enhance self-image scores, and offer additional autograft for future surgeries [90,91,92,93,94,95,96,97]. However, the utilization of TP is surrounded by controversy. Kumar et al. [95] concluded that the overall decrease in pulmonary function after thoracoplasty necessitates the need for adequate pre-operative pulmonary function to mitigate its effect on patient well-being. The use of a posterior approach for corrective surgery when thoracoplasty is planned might lead to better outcomes. Other research has indicated inconclusive outcomes of TP on pulmonary function tests, with documented complications including respiratory insufficiency, parietal pleura tear, and subsequent pleural effusion or pneumothorax [96,97,98,99]. Figure 12 presents 14-year-old female patient suffering from severe adolescent idiopathic scoliosis underwent treatment involving pre-operative halo gravity traction, followed by multi-level Ponte osteotomies, a rib resection with thoracoplasty procedure, and PSF.

A meta-analysis conducted by Turner et al. [97] compared pulmonary function in adolescent idiopathic scoliosis patients undergoing posterior spinal fusion with and without thoracoplasty. They observed a notable decline in %FVC after Posterior Spinal Fusion with Thoracoplasty and a slight, yet significant enhancement in %FEV1 after Posterior Spinal Fusion alone. Another meta-analysis by Lee et al. [35] discussed alterations in absolute pulmonary function tests (PFTs) after corrective surgery. They noted that anterior spinal fusion and instrumentation led to a moderate to large decrease in absolute PFTs within the initial three months post-surgery, which normalized after 2 years. Additionally, they found that posterior spinal fusion without thoracoplasty had mild to moderate favorable effects on PFTs. Notably, reducing thoracic complications associated with convex TP can be achieved through refining surgical techniques and employing respiratory function monitoring [96,97]. Other studies [98,99] have demonstrated that pulmonary function post-thoracoplasty not only reaches pre-operative levels, but significantly surpasses them with regards to the majority of the pulmonary parameters measured; furthermore, satisfactory radiological correction and clinical outcomes were also demonstrated. Pulmonary arterial hypertension, ventricular diastolic function, and pulmonary function were improved after halo-pelvic traction and thoracoplasty. A moderate negative correlation has been identified between pulmonary artery pressure and pulmonary function: as pulmonary function improved, pulmonary artery pressure decreased [54]. Correction of rib hump in scoliosis surgery plays a crucial role in enhancing patient satisfaction. Thoracoplasty not only enhances aesthetics, but also offers an additional source of autologous bone graft while having a minimal impact on pulmonary function over a 2-year follow-up period. Despite advancements in vertebral derotation techniques, thoracoplasty continues to be relevant due to its associated benefits. Although thoracoplasty is a gratifying procedure (i.e., linked to increased satisfaction levels post-surgery according to self-image assessments), it is not without risks, including temporary pulmonary function impairment, chest wall pain, potential chest drainage complications, flail chest, and pseudoarthrosis. A new strategy has been developed to address the moderate and severe rib hump deformities in scoliosis patients, with a focus on the benefits of osteosynthesis post-partial rib resection. This innovative approach, known as Thoracoplasty Reconstruction with Internal Osteosynthesis (TRIO), involves stabilizing the rib stumps through rib clips following partial costectomy. The TRIO technique offers potential advantages, such as improved correction of rib prominence, reduced postoperative pulmonary function impairment, decreased chest wall pain, and lower incidence of rib pseudoarthrosis. In a study with 5-years of follow-up, the authors noted that, regardless of whether thoracoplasty was performed or not, FVC, FEV1, and expiratory flow were improved 5 years or later after PSF [100]. This study presented findings from a prospective investigation into the effectiveness of a modified Concave Rib Osteotomy (CRO) technique combined with posterior instrumented fusion for the treatment of severe and inflexible curves in adolescent idiopathic scoliosis patients. The average correction in the frontal plane after surgery was 68%, while the incidence of pulmonary complications stood at 11.5%. The altered CRO technique has been shown to be a secure option in lieu of anterior release for severe and rigid curves [100]. Posterior spinal releases in non-scoliotic spines led to a gradual rise in spinal flexibility, although subsequent steps had diminishing impact. In comparison to SIL resection with inferior facetectomy, the addition of superior facetectomy did not enhance flexibility in AR and LB, with only a 6.3% increase in flexion. It is important to exercise caution when interpreting the data from this in vitro study, as no suitable cadaveric spine model for AIS was available. Nevertheless, the findings here raise doubts regarding the advantages of routinely conducting complete facetectomies (e.g., Ponte osteotomies) to enhance spinal flexibility in scoliosis surgery [77]. An adjusted method for thoracoplasty was effective and secure in rectifying razor back deformities in patients with severe, rigid scoliosis and severe pulmonary issues, without causing notable changes in their long-term pulmonary function [93,94]. Pairing thoracoplasty with AIS surgery enhances the self-image of patients without impacting post-operative PFTs at the 2-year mark. Given the low risk involved in thoracoplasty, it is sensible to offer this option to individuals with pronounced rib hump and high aesthetic expectations [29]. The inclusion of thoracoplasty in AIS surgeries did not lead to increased immediate post-operative pain, narcotic usage, or oxygen consumption when combined with PSF. Enhanced SRS self-image scores were observed following thoracoplasty [27]. Posterior correction surgery for mild to moderate AIS individuals did not result in significant enhancement of post-operative respiratory function, as measured through relative, percent-predicted values at a minimum 2-year follow-up [53]. Employing bilateral costoplasty alongside scoliosis correction could offer a secure and effective approach for managing severe rib cage deformities linked to thoracic scoliosis. This method should be considered in cases with prominent rib hump deformities, where correction through scoliosis correction alone or with unilateral costoplasty may not be sufficient [28]. In the context of thoracoplasty performed on individuals with severe scoliosis and razor back deformity, the average predicted forced vital capacity (FVC%) prior to the surgical intervention was recorded at 37.2 ± 13.30% [81]. This value is reflective of a substantial degree of pulmonary impairment, signaling the presence of significant respiratory challenges. Interestingly, a marginal increase was observed in the FVC% during the final assessment, although this change did not reach statistical significance. Moreover, the mean total lung area presented an increase from 2583.2 ± 501.36 to 2890.1 ± 537.30 mL at the conclusion of the follow-up period, indicating a positive trend in lung capacity. It is worth noting that no instances of severe pulmonary complications were documented throughout the course of treatment. The overall execution of the thoracoplasty procedure demonstrated effectiveness and safety in addressing the razor back deformity in patients characterized by severe and inflexible scoliosis, alongside pronounced pulmonary dysfunction. Importantly, this intervention did not elicit any notable alterations in the long-term pulmonary function of the individuals involved in the study [81,94,95,96,97,98]. The comparison of all analyzed techniques are placed in Table 1.

## 11. Author’s Preferred Technique

Based on a comprehensive examination of the existing medical literature regarding the management of severe scoliosis, coupled with our vast expertise in performing surgical interventions for scoliosis exceeding 100 degrees in pediatric and adolescent populations, we aim to outline our treatment approach. Initially, the primary objective of addressing severe spinal deformities is ensuring the safety of the patient. When confronted with severe and rigid deformities that are frequently overlooked, it is essential to strike a balance between our surgical objectives and the aspirations of the patient and their family. Given that complications arising from the treatment of severe scoliosis can lead to catastrophic outcomes, our fundamental philosophy revolves around minimizing the occurrence of such complications to the greatest extent feasible. It is a well-established fact in the literature that the duration of the surgical procedure directly correlates with the likelihood of complications, as prolonged surgical interventions can compromise the surgeon’s focus and the precision of their decisions and techniques, consequently increasing the risk of complications. Conversely, patients with substantial curvature often exhibit compromised respiratory function, nutritional deficiencies, and an overall decrease in physical capabilities. Thus, our methodology is founded on the concept of incremental progress towards the ultimate objective, rather than rushing towards the finish line like a sprinter.

Primarily, our approach entails enhancing respiratory function and overall bodily efficiency gradually and steadily, rather than hastening the process, through the application of HGT therapy. This method, which is cost-effective and associated with an acceptable level of risk for complications, typically involves a six-week period of traction combined with intensive rehabilitation and physical therapy. In cases of severe patients, extending the traction period to six months can yield remarkable outcomes, facilitating a safer surgical intervention, particularly in patients afflicted with conditions such as osteogenesis imperfecta. Following the preparatory phase, the surgical procedure involves a multi-level release of the spine, mobilization for correction utilizing a multi-level Ponte osteotomy, a technique validated by studies referenced previously. Additionally, excising a prominent rib hump at the apex of the curvature not only produces a cosmetic enhancement but also enhances the spinal correction potential. In instances where the combined duration of these techniques is anticipated to exceed 6–7 h, the procedure is divided into two stages, with each stage performed several days to three weeks apart [22,24,44].

For patients unable to undergo HGT traction due to various reasons or those who decline this treatment modality, we promptly initiate a staged intervention utilizing our innovative approach of less invasive temporary internal distraction. This two-stage process involves a multilevel Ponte osteotomy split into two operations, incorporating spinal distraction under the monitoring of neuromonitoring systems, yielding excellent and secure outcomes as corroborated by the previously cited publications [22,24,44]. Furthermore, during the second stage, a less invasive thoracoplasty procedure may be conducted to further enhance the treatment outcomes. Techniques involving vertebral resection are exclusively reserved for cases of bone-related scoliosis or following numerous revision surgeries, such as in situations necessitating spondylodesis [30,31].

In the realm of patient safety, the operational trajectory, and potential complications, the issue of intraoperative blood loss stands out as a pivotal concern. Within the comprehensive treatment course for severe scoliosis, we contend that this particular matter holds significant weight. Our modus operandi and procedural benchmark revolve around the utilization of Tranexamic Acid (TXA) in a continuous infusion, adhering steadfastly to the recommended dosages throughout the entirety of the surgical procedure. Right from the inception of incision, the patient is carefully maneuvered under the vigilant supervision of our seasoned anesthesiologist, ensuring a negative pressure environment typically set at 70–80 mmHg. This meticulous approach not only aids in curtailing blood loss during initial access but also facilitates the creation of necessary channels for screw placement. In the intricate phase of Ponte osteotomy, we augment our strategy by incorporating local hemostatic agents to further bolster hemostasis. The incorporation of a cell saver system is highly coveted as it proves instrumental in minimizing overall blood loss. Moreover, the employment of bone wax emerges as a highly effective technique in stemming bleeding from bone surfaces, thereby contributing significantly to the overall reduction of blood loss. A favorable option is to use a bone knife, which reduces bleeding. Subsequent to the insertion of screws and rods into their designated positions, just prior to the corrective maneuvers, the overseeing anesthesiologist deliberately elevates the patient’s blood pressure beyond 100 mmHg. This preemptive measure is aimed at averting spinal cord ischemia during the correction phase and warding off the onset of any potential neurological deficits. The administration of TXA via a pump continues unabated until the closure of the surgical wound is completed, ensuring a sustained therapeutic effect [101,102,103,104,105,106,107,108].

**Figure 13:**
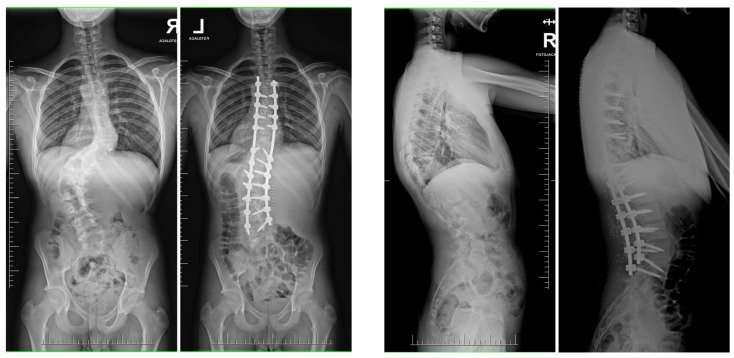
An example of a 16-year-old boy with congenital scoliosis treated with posterior-only VCR and PSF followed by multi-level Ponte osteotomies, and eventual correction using double Co-chr 6.0 rods. Radiological photographs were taken both before and after the surgical intervention, as part of the observation period.

Evidence derived from numerous scientific studies suggests that patients undergoing non-vertebral column resection (non-VCR) surgical procedures are significantly more safe with less complications, than at a rate about 7 times higher, to encountering neurological complications in comparison to those undergoing VCR surgical techniques. Figure 13 showed an example of a 16-year-old boy with congenital scoliosis treated with posterior VCR and PSF. This finding underscores the importance of opting for less drastic surgical approaches when managing severe cases of scoliosis or kyphoscoliosis. Moreover, when coupled with HGT (growth-friendly techniques), utilizing these less aggressive surgical methods can lead to comparable and satisfactory outcomes in the correction of major spinal curves among these patient populations [11].

## 12. Conclusions

Halo traction, halo-femoral traction, and techniques for spine release (e.g., anterior, posterior, or combined) have demonstrated significant effectiveness in managing severe and rigid idiopathic scoliosis and remain the gold standard for the treatment of severe spinal deformities. The combination of several of these methods can lead to optimal alignment correction without the need to perform high-risk techniques for three-column osteotomies. As such, surgeons must customize the indications based on factors such as available resources, characteristics of the deformity, and the patient’s individual profile. Surgical correction of severe scoliosis without vertebral body resection surgeries decreases the potential risks of neurological and pulmonary complications while providing significant clinical improvement outcomes; therefore, they also should be considered and performed by a proficient surgical team. The powerful Ponte osteotomy is indicated for severe scoliosis, curves with poor flexibility, for better restoration of hypokyphosis and decrease of hyperkyphosis. These corrective techniques combined with HGT or temporary internal distraction rods are recommended as viable options for managing individuals with severe rigid spine deformity characteristics. Notably, the presence of neuromonitoring is crucial throughout these procedures.

## Figures and Tables

**Table 1:** The comparison of all analyzed techniques and outcomes in relation to the main curve correction are summarized. MCGR, magnetically controlled growing rod; HGT, halo gravity traction; PSF, posterior spinal fusion; VCR, vertebral column resection; HFT, Halo-femoral traction; TID, temporary internal distraction; NA, not applicable/available.

Study	Technique Used	Mean Preoperative Cobb, Degree	Mean Preoperative Flexibility (%)	Correction (%) of Main Curve	Mean Postoperative Cobb, Degree
Koller et al. [16]	HGT + PSF	106	10	35	70
Watanabe et al. [17]	HGT + PSF	119	20	46	58
Rinella et al. [37]	HGT + PSF	84	NA	46	38
Rocos et al. [38]	HGT + PSF	123	NA	49	61
Bogunovic et al. [39]	HGT + PSF	96	NA	61	63
Garabekyan et al. [43]	HGT + PSF	101	NA	43	58
Grabala et al. [44]	HGT + Ponte osteotomies + PSF	118	Less than 30	65	42
Grabala et al. [44]	MCGR TID + Ponte osteotomies + PSF	112	Less than 30	61	43
Mehlman et al. [51]	Spinal release and HFT + PSF	95	29	71	32
Takeshita et al. [62]	Intra-operative traction + PSF	87	NA	59	35
Hamzaoglu et al. [58]	Intra-operative HFT + PSF	122	39	51	60
Qiu et. Al. [57]	Anterior release + HFT + PSF	96	23	45.2–57.5	57
Potaczek et al. [65]	HFT + PSF	116	21	45	61
Grabala et al. [22]	HGT + Ponte osteotomies + PSF	124	18	63	45
Grabala et al. [22]	Less invasive TID + Ponte osteotomies + PSF	122	21	70	37
Buchowski et al. [23]	TID + Ponte osteotomies + PSF	104	16	80	20
Skaggs et al. [33]	TID + PSF	113	26	54	62
Hu et al. [79]	TID + PSF	148	Less than 15	46	79
Koller et al. [81]	MCGR TID + Ponte osteotomies + PSF	118	20	67	38
Di Silvestre et al. [83]	MCGR TID + Ponte osteotomies + PSF	98	25	59	38
Sponseller et al. [21]	VCR + PSF	94	NA	66	38
Van Halm-Lutterodt et al. [26]	Ponte Osteotomy + PSF	More than 80	Less than 25	57	NA
Van Halm-Lutterodt et al. [26]	Smith Petersen-Osteotomy + PSF	More than 80	Less than 25	49	NA
Stone et al. [34]	Anterior release + PSF	112	17	66	38
Stone et al. [34]	VCR + PSF	126	23	62	70
Ren et al. [49]	VCR + PSF	101	23	67	51
Ren et al. [49]	Anterior release, TID + PSF	104	22	75	51
Zhou et al. [50]	VCR + PSF	99	12	65	33
Bullmann et al. [4]	Anterior release + PSF	93	23	67	31
Potaczek et al. [65]	Anterior release + PSF	108	21	51	54
Suk et al. [67]	PSF	79	44	70	24
Potaczek et al. [65]	PSF	112	25	38	63
Shen et al. [68]	Staged: anterior/posterior	99	31	57–59	39–41
Potaczek et al. [65]	Anterior release + HFT + PSF	125	20	52	62
El Masry et al. [100]	Concave rib osteotomy + PSF	91	24	68	29.5

## Data Availability

The data are contained within this article.
